# Correspondence

**Published:** 1904-03

**Authors:** 


### CORRESPONDENCE.

The following has been received from Messrs. Parke, Davis & Co.:

THE JOURNAL OF THE AMERICAN MEDICAL ASSOCIATION.
103 DEARBORN AVE., CHICAGO, January 15, 1904.

*Messrs. Parke, Davis & Co., Detroit, Mich.*

GENTLEMEN: There has been a good deal of comment in the newspapers in reference to the alleged combination of the antitoxin manufacturers, with a consequent rise in price, which it is claimed is 50 per cent. over former prices. How much truth is there in this statement? Kindly let me have your side, as we have been requested to take up the matter. Also, will you kindly send me your price-list in force three months ago or more, and that of to-day, both wholesale and retail.

Thanking you in advance for the favor of a reply, I am,

Respectfully yours,

GEORGE A. SIMMONS, Editor.

---

January 16, 1904.

*George H. Simmons, M.D., Editor of the Journal of the American Medical Association, 103 Dearborn Avenue, Chicago, Ill.*

DEAR SIR: In answer to your inquiry of the 15th we have pleasure in making out the following statement.

Beginning January 1, 1904, we market only one grade of antitoxin, in four packages or doses:

| Number of Units. | List Price |
|---|---|
| 1000 | $2.00 |
| 2000 | 3.50 |
| 3000 | 5.00 |
| 4000 | 6.50 |

*Trade Discounts:* 25 per cent. to the retail, and $33\frac{1}{3}$ per cent. to the wholesale druggist.

*Injecting Device:* With each package of the "new serum" we now supply an expensive device for administering the contents, sparing the physician the need of buying and sterilizing a serum syringe.

*Last Year's Prices:* Last year we marketed *two* grades of Anti-diphtheritic Serum—the Standard or X serum, testing 200 and more units to the Cc.; the more powerful and concentrated Special or XX, testing 500 and more units to the Cc.,—at the following prices:

| Number of Units. | X Prices. | XX Prices. |
|---|---|---|
| 1000 | $1.50 | $2.25 |
| 2000 | 3.00 | 4.00 |
| 3000 | 4.50 | 5.75 |
| 4000 | Not formerly listed. | Not formerly listed. |

*Last Year's Discounts:* Only 20 per cent. to the retailer; to the wholesaler, 33⅓ per cent.

*Injecting Devices:* None furnished until the close of the year.

*Potency of the New Serum:* In the 4000-unit package we are placing serum testing on an average 600 units to the Cc.; in the 3000-unit package, serum testing on an average 500 units to the Cc.; in the 2000-unit package, serum testing on an average 400 units to the Cc.; and in the 1000-unit package, serum testing on an average 300 units to the Cc.

Thus, comparing prices and discounts, old and new, allowing for the additional cost of the injecting device, and remembering that the new serum, in point of concentration and antitoxic potency, is much superior to the X, and all but equal to the XX, you will see that our 1904 prices *exhibit a reduction and not an advance.*

*Free Exchanges: Fully forty per cent. of all the serum we sell comes back for free exchange.* What becomes of the returned serum? *It is poured into the sewer.* This is the sole reason for our marketing henceforth one grade of antitoxin in place of two, and *four* packages or doses in place of ten. The producer, the retailer, and the wholesaler will be spared a useless trouble and an enormous expense, while the physician *loses nothing* thereby.

*The Chicago Department of Health:* For years the department profited by the illegal and unconstitutional action of the New

York Board of Health, which sold to Chicago its surplus serum. When Mayor Low and Dr. Lederle discontinued such sale, the Department, by playing off one producer against another, succeeded in screwing down the price until its business was altogether unprofitable. We *know* that every sale of serum *ever* made the Chicago Department by a private producer has been made at a loss. We see no reason why, in buying antitoxin for its poor, the great city of Chicago should compel us to furnish our product at a loss. How much money will Chicago have to spend for antitoxin to be donated to its poor? Less than $5,000.00 a year. If a single life is sacrificed among the indigent sick of your city, it will be solely because the Department refuses to pay a fair and reasonable price for a good product. *As for the diphtheria sufferers who are not charity patients,* they will henceforth pay *less and not more,* quality considered.

*Federal Supervision:* A federal law compels all makers of biological products to work under federal *license and supervision,* the license being revocable for faulty equipment of men or appliances, or unsatisfactory product.

Considering the harassing dangers and responsibilities of our biological work, our immense investment of capital, our employment of the most expert scientific talent, and the heavy waste in the form of free exchanges, we feel wholly warranted in writing and urging you to send to Detroit, at our expense, a trusty and and accomplished biologist. Let him come, see our laboratories, compute our expenses and producing costs, and publish his report in your pages. We are prepared to stand or fall by the verdict of any unbiased expert or commission.

Very truly yours,

